# Detection and Phenotypic Antimicrobial Susceptibility of *Salmonella enterica* Serotypes in Dairy Cattle Farms in the Po Valley, Northern Italy

**DOI:** 10.3390/ani14142043

**Published:** 2024-07-12

**Authors:** Francesca Parolini, Giordano Ventura, Carlo Rosignoli, Sara Rota Nodari, Mario D’incau, Leonardo Marocchi, Giovanni Santucci, Massimo Boldini, Matteo Gradassi

**Affiliations:** 1 Istituto Zooprofilattico Sperimentale della Lombardia e dell’Emilia Romagna, 26100 Cremona, Italy; giordano.ventura@izsler.it (G.V.); massimo.boldini@izsler.it (M.B.); 2 Istituto Zooprofilattico Sperimentale dell’Abruzzo e del Molise “G. Caporale”, 64100 Teramo, Italy; f.parolini@izs.it; 3 Istituto Zooprofilattico Sperimentale della Lombardia e dell’Emilia Romagna, 46100 Mantova, Italy; carlo.rosignoli@izsler.it (C.R.); leonardo.marocchi@izsler.it (L.M.); giovanni.santucci@izsler.it (G.S.); 4 Istituto Zooprofilattico Sperimentale della Lombardia e dell’Emilia Romagna, 25124 Brescia, Italy; sara.rotanodari@izsler.it (S.R.N.); mario.dincau@izsler.it (M.D.)

**Keywords:** *Salmonella* spp., salmonellosis, animal health, dairy cattle, antimicrobial resistance

## Abstract

**Simple Summary:**

Salmonellosis represents a considerable public and animal health concern. Cattle and particularly calves can be affected by this infection, which can cause a variety of clinical problems. At present, data about the diffusion of *Salmonella* spp. in dairy cattle farms of northern Italy are scarce. In this context, this study aims to conduct a retrospective survey on the presence of *Salmonella* serotypes in dairy cattle farms in the Cremona and Mantua provinces of the Lombardy Region (northern Italy). The results highlight that in most cases, *Salmonella* spp. was detected in the carcasses or organs of calves and that its presence was widely distributed on the farm. Finally, the analyses of antimicrobial resistance patterns place the attention on the spread of multi-drug resistant *Salmonella* strains.

**Abstract:**

The presence of *Salmonella* spp. in dairy cattle farms poses a major risk to animal health and welfare. This study focused on *Salmonella* detection in dairy farms located in the Cremona and Mantua provinces (northern Italy) in samples collected and submitted to laboratories in 2021–2022. A total of 2710 samples from different sources, including calf carcasses/organs (*n* = 128), rectal swabs (*n* = 1937), feces (*n* = 390), bulk milk (*n* = 93), and overshoes/swabs (*n* = 127) for environmental sampling, were analyzed for the presence of *Salmonella* spp. and were included in the present study. Our results indicate that *Salmonella* was most commonly firstly identified from calf carcasses and organs (61.67%) and that the serotypes most frequently detected in dairies were *S*. Dublin (38.33%), *S*. Typhimurium (23.33%), and *S*. Typhimurium monophasic variant (14.17%). The most common pathological findings in calf carcasses were enteritis, hepatosplenomegaly, and pneumonia. The antimicrobial resistance pattern analyzed using the MIC assay of 51 *Salmonella* isolates revealed the presence of multi-resistant strains, which pose a major risk to public and animal health.

## 1. Introduction

Cattle farms are not evenly distributed throughout the Italian territory, but the highest density occurs in the Po Valley, North Italy. Almost 30% of the whole Italian cattle population is farmed in the territory of the Lombardy Region, where dairy farms with the highest herd size are located. Notably, this region accounted for 45.4% of the national milk production in 2021–2022 (AGEA data for 2022). In this scenario, animal health, animal welfare of dairy cattle, and food safety for consumers are of key importance.

*Salmonella* is a Gram-negative ubiquitous bacterium that belongs to the family of *Enterobacteriaceae*. To date, two different *Salmonella* species have been identified—*S*. *bongori* and *S*. *enterica*. The last one comprises different subspecies, among which *Salmonella enterica* subsp. *enterica* is of the highest interest in human and veterinary medicine [1,2]. *Salmonella* can be introduced onto a dairy farm by livestock, feed, water, fertilizer, insects, wildlife, people, or equipment. Otherwise, the bacteria may be present in healthy carrier animals that, in stressed conditions, can shed *Salmonella* in feces, contaminating the environment. Indeed, the primary transmission route of *Salmonella* is the fecal–oral one [3,4]. Following ingestion, *Salmonella* can colonize the intestinal tract and then create an infection [5]. The disease development can occur in both calves and adult cattle, although the most frequent clinical forms are reported in young calves, in which it may be fatal. Various clinical forms are associated with *Salmonella* infection, such as enteric, septicemic, pulmonary, and reproductive diseases; all are possible manifestations of salmonellosis [3]. In Europe, *S*. Dublin, the host-adapted serovar for cattle [6]; *S*. Typhimurium; and monophasic *S*. Typhimurium (1,4, [5], 12: i:-) are the most frequently isolated serovars in cattle [7]. Different serovars result in variations in the symptomatology of infection, as each one has specific virulence factors [5]. *S*. *enterica* serovar Typhimurium is commonly associated with enteric disease in calves that are less than two months of age, while *S*. *enterica* serovar Dublin is correlated to disease in young and adult cattle. *S*. Dublin is more invasive than *S*. Typhimurium, and it can be responsible for a wide spectrum of lesions, such as meningoencephalitis, splenomegaly, hepatitis, cholecystitis, serositis, and arthritis, with or without signs of enteric disease [5,8]. Moreover, *S*. Dublin has a propensity to cause respiratory problems in older calves [9].

At present, in Italy, a national surveillance program to monitor *Salmonella* status in cattle is not available. *Salmonella* spp. are included in the list of zoonotic agents that need to be monitored for public health, according to the 2003/99/CE [10]. *Salmonella* is considered a major food safety concern, as the main risk of *Salmonella* transmission to humans is associated with the consumption of contaminated food [7]. In addition, salmonellosis can be a costly disease because of increased culling rates, mortality, treatment costs, reduced feed efficiency, and decreased weight gain and milk production [11]. For these reasons, it is crucial to prevent *Salmonella* outbreaks in dairy herds and to develop appropriate measures and preventive actions to counteract it. Once *Salmonella* infection has been detected and confirmed in dairy farms, several control measures can be implemented. Prevention strategies include good biosecurity; preventing pathogen introduction into the farm (external biosecurity), as well as pathogen transmission on the farm itself (internal biosecurity); appropriate effluent management; and severe hygiene practices. In addition, the prevention of salmonellosis is possible through vaccination. Several *Salmonella* vaccines, including bacterins, modified live subunits, and attenuated vaccines, have been evaluated in calves [12]. Commercial vaccines for *Salmonella* spp. in cattle are not licensed in Italy. Instead, inactivated autogenous vaccines can be produced on demand. Another weapon available to counter *Salmonella* infection is antimicrobial therapy. Although antimicrobial administration is sometimes necessary to aid in clinical recovery, the scientific and medical communities recommend their prudent use, paying attention to any resistance evidenced through in vitro susceptibility tests. In this scenario, the study of the antimicrobial resistance (AMR) in bacterial pathogens is crucial. Both phenotypic and genotypic studies can be performed to evaluate AMR, using minimum inhibitory concentration (MIC) and whole genome sequencing (WGS), respectively. Furthermore, WGS has been vital for revealing the rapid temporal and spatial evolution of antimicrobial resistance (AMR). For *Salmonella*, tetracyclines have already achieved high levels of resistance globally, leaving the conservation of other antimicrobials to be of particular importance [13,14,15].

The present study aims to conduct a retrospective analysis on the detection of *Salmonella* spp. in dairy cattle farms of the Cremona and Mantua provinces of the Lombardy Region (Northern Italy) and to evaluate the phenotypic antimicrobial susceptibility profile among the *Salmonella* serotypes isolated. Our results fill the knowledge gap about the presence and diversity of *Salmonella enterica* serovars in dairy farms in the study area, also providing information about the antibiotic resistance profiles of *Salmonella* spp., as well as the *Salmonella* distribution on the farm.

## 2. Materials and Methods

### 2.1. Study Area and Cattle Population

This retrospective study covered a two-year period between January 2021 and December 2022. The study area included the two provinces of Cremona and Mantua in the Lombardy Region, where 1068 dairy cattle farms with a population of nearly 350,000 cattle were present during the study period (Banca Dati Nazionale 2022).

### 2.2. Sample Collection

This study was carried out on samples that were delivered for diagnostic purposes to the Diagnostic Laboratories of Istituto Zooprofilattico Sperimentale della Lombardia e dell’Emilia Romagna (IZSLER) of Cremona (CR, Italy) and Mantua (MN, Italy).

Dairy farms, with at least one *Salmonella* isolation in the studied period, either with a clinical outbreak of the disease or with subclinical infections, were included in the study. After the first isolation of *Salmonella*, 60 farms were subjected to further sampling to monitor the spread of infection and to evaluate the effectiveness of the control measures implemented on the farm. Specifically, the samples collected during the control period were calf feces and rectal swabs; calf carcasses/organs and fetuses; bulk milk; and environmental samples such as overshoes and swabs/sponges.

Rectal swabs and feces from individual calves (<one month of age) were taken to identify *Salmonella* shedders prior to the animals’ movement. When antimicrobial therapy was carried out on calves, sampling was carried out at least seven days after the end of the treatment in order to reduce the risk of false-negative results.

Environmental absorbent socks worn over shoes (overshoes) were taken from different areas of the farm, in order to monitor the spread of *Salmonella* and the contamination of the environment; environmental swabs/sponges were collected from individual calf pens at the end of the cleaning and disinfection protocol in place at the farm to verify their effectiveness.

### 2.3. Pathological Examination

Autopsies were performed on deceased animals, and the macroscopic lesions observed during necropsy were recorded. Selected tissues and fluid samples from carcasses were aseptically collected at autopsy for microbiological culture.

### 2.4. Microbiological Analyses

The isolation of *Salmonella* spp. from different sources was carried out according to the International Organization for Standardization (ISO): ISO 6579-1:2017/Amd 1:2020 [16] method for detection of *Salmonella* spp.

### 2.5. Serogroup and Serotype Identification

*Salmonella* spp. serogroup and serotype identification was performed according to ISO/TR 6579-3:2014 [17]. For the serological characterization of *Salmonella*, slide agglutination and tube agglutination methods were used for the determination of somatic and flagellar antigens, respectively [18,19]. The results of the antigen determinations were used for the final serological characterization according to the scheme of Kauffmann–White–Le Minor [20].

### 2.6. Antimicrobial Susceptibility Testing

The antimicrobial susceptibility of obtained isolates was tested using the MIC assay using commercially prepared panels with the following antibiotics: aminosidine (1–32 µg/mL, AN), amoxicillin + clavulanic acid (0.25–0.12 µg/mL, AMC), ampicillin (0.25–32 µg/mL, AMP), cefazolin (0.5–8 µg/mL, CFZ), cefotaxime (0.5–4 µg/mL, CTX), colistin (0.03–8 µg/mL, COL), enrofloxacin (0.015–32 µg/mL, ENR), florfenicol (1–64 µg/mL, FLO), flumequine (1–16 µg/mL, FLU), gentamicin (0.25–32 µg/mL, GEN), kanamycin (2–32 µg/mL, KAN), sulfisoxazole (128–515 µg/mL, SFX), tetracycline (0.5–16 µg/mL, TET), and trimethoprim + sulfonamides (0.06/1.19–16/304 µg/mL, SX-T) (SensititreTM ITISVE1, Thermo Fisher Scientific, Waltham, MA, USA). As determined by the reference center for antibiotic resistance (Istituto Zooprofilattico Sperimentale del Lazio e della Toscana, Centro di Referenza Nazionale per l’Antibiotico resistenza, CRAB, Rome, Italy), the antimicrobials tested in the MIC panel were ‘class representative’ for different classes or subclasses of antimicrobials. MICs were interpreted using CLSI VET08 [21] standards for amoxicillin + clavulanic acid, ampicillin, cefazolin, florfenicol, gentamicin, kanamycin, tetracycline, and trimethoprim + sulfonamides. CLSI–M100 [22] was used for the breakpoints for cefotaxime and sulfisoxazole. The breakpoints reported in CA–SFM [23] and SFM-VET [24] were used to estimate resistance for aminosidine (MIC ≤ 8 mg/L susceptible; MIC > 16 mg/L resistant) and flumequine (MIC ≤ 4 mg/L susceptible; MIC > 8 mg/L resistant), respectively. The MIC values for colistin and enrofloxacin were interpreted using breakpoints reported by EUCAST [25]. Quality control testing was performed on *Escherichia coli* ATCC 25922 and *Staphylococcus aureus* ATCC 29213.

### 2.7. Autogenous Vaccine Development

*Salmonella*-inactivated autogenous vaccines were produced from the *Salmonella* strains isolated in affected farms by the Vaccine and Reagent Production Laboratory at IZSLER, following Legislative Decree no. 287 of 17 March 1994. The autogenous vaccines were produced for each farm using the serotypes isolated in that specific farm. Briefly, each *Salmonella* isolate was cultured at 37 °C in a bio fermenter and was inactivated with 37–40% formaldehyde (0.8%/culture volume). The autogenous vaccine was prepared at a concentration of 7 billion/mL of inactivated bacteria and was adjuvated with aluminum hydroxide. The vaccination program involved administering the primary doses (3 mL) subcutaneously in the cow’s neck region and the subsequent booster vaccination doses 21–28 days later.

### 2.8. Data Analysis

Data plotting was conducted with GraphPad Prism 8.2 (GraphPad Software Inc., La Jolla, CA, USA) and Microsoft Excel 2016. Because of the retrospective nature of this study, in which all samples were voluntary conferred to the laboratories for diagnostic purposes, the selection bias introduced by the non-probabilistic nature of the sampling forbids the application of inferential approaches. Therefore, only the proportions of positive samples were calculated.

## 3. Results

### 3.1. Overall Samples

**Figure 1:**
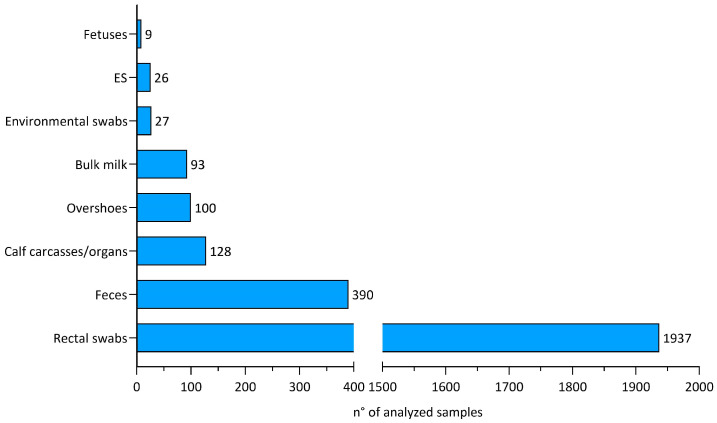
Overall samples analyzed for *Salmonella* spp., according to the sample type.

A total of 120 cases of *Salmonella* infection from 118 dairy cattle farms were reported in the study period, with 61 cases in 2022 and 59 in 2021. Of note, two herds were involved in repeated cases caused by different *Salmonella* serovars in two consecutive years. The overall number of samples analyzed for *Salmonella* spp. (*n* = 2710) divided by sample type is shown in Figure 1. The majority of the samples were rectal swabs from calves (*n* = 1937), followed by calf feces (*n* = 390), calf carcasses/organs (*n* = 128), and environmental overshoes (*n* = 100).

### 3.2. Isolated Salmonella Serotypes

*S*. Dublin, *S*. Typhimurium, and its monophasic variant (1,4, [5], 12:i:-) were the most commonly isolated serotypes, accounting for 75.83% of the total. Overall, 17 different serotypes were detected and their prevalence is shown in Table 1. In most cases, only one *Salmonella* serovar was detected in the farm (107/120), while in 13 cases (10.8%), simultaneous infection with two or more serotypes was found. Interestingly, in two farms, three different serovars of *Salmonella* were isolated—*S*. Dublin, *S*. Typhimurium, and *S*. Typhimurium monophasic variant in one case, and *S*. Agama, *S*. Typhimurium, and *S*. Typhimurium monophasic variant in the other.

### 3.3. First Isolation Sources

**Figure 2:**
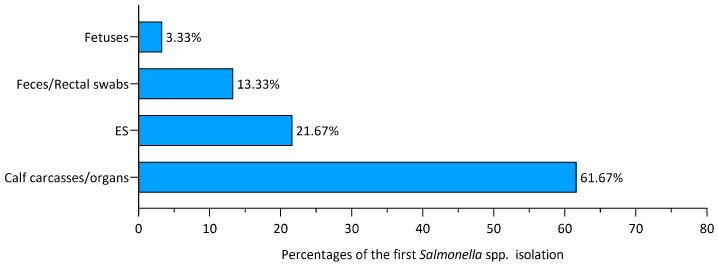
Percentages of the first *Salmonella* isolations in the farm according to their source. ES: emergency slaughter samples.

From the analysis of our data, it was found that in the majority of cases (74/120, 61.67%), the first detection of *Salmonella* in the herd occurred after autopsy on calf carcasses/organs conferred to the laboratories for diagnostic purposes. In the case of suspected *Salmonella* infection, microbiological research was performed from different tissue samples such as gut, mesenteric lymph nodes, spleen, gallbladder, liver, and lung. Furthermore, microbiological analyses of feces/rectal swabs and fetuses led to the finding of *Salmonella* in a minority of cases, respectively, 16/120 (13.33%) and 4/120 (3.33%). Of note, 21.67% of cases (26/120) were derived from the isolation of *Salmonella* from cows’ liver samples collected by official veterinarians during post-mortem inspection after emergency slaughter carried out at the farm. Figure 2 shows the different sources of first isolation and their percentages.

### 3.4. Anatomopathological Examination

The autopsies of 91 naturally deceased calves (under one-year in age) were performed, and the macroscopic lesions were recorded. The macroscopic lesions observed in animals infected with different *Salmonella* serotypes are summarized in Table 2. 

### 3.5. Follow-Up Analyses

After the first detection of *Salmonella*, additional samples were collected, with the collaboration of veterinary officers, field veterinary practitioners, and farmers in 60 farms (61 out of 120 cases) and were analyzed for the presence of *Salmonella* to assess the spread of *Salmonella* within the farm and to evaluate the effectiveness of the control measures implemented.

The total number of analyzed samples (*n* = 2405) and the proportion of positive samples are listed in Table 3. Among them, feces/rectal swabs were the most represented (*n* = 2145), followed by environmental overshoes (*n* = 100), bulk milk (*n* = 93), calf carcasses/organs (*n* = 36), environmental swabs/sponges (*n* = 27), and aborted fetuses (*n* = 4).

Rectal swabs and feces analysis were performed to detect *Salmonella* shedding in the feces of young calves (< one month old) and yielded a positive result in 206 out of 2145 samples (9.6%).

Overshoes have been used for the environmental sampling of different farms areas. *Salmonella* was isolated in 34 out of 100 overshoes (7/16 farms), with the following proportion of positivity: collective pens for calves (22/61), maternity area (12/33), lactation group area (0/4), and dry period area (0/2). 

Environmental swabs/sponges on the individual pens for calves were taken after the cleaning and disinfection procedures were implemented in the farm and yielded a *Salmonella* positive result in 9 out of 27 samples (4/7 farms).

Regarding bulk milk surveillance, 93 bulk milk samples from 32 farms were analyzed, of which three tested positive for *Salmonella* (3/32 farms).

### 3.6. Antimicrobial Susceptibility Testing

Fifty-one non-repetitive isolates of *Salmonella* spp. were subjected to the Minimal Inhibitory Concentration (MIC) assay, to determine antibiotic susceptibility. Serovars of the analyzed isolates are reported in Table 4. 

These *Salmonella* isolates displayed a variable spectrum of antibiotic resistance. Only 3.92% (2/51) strains (*S*. Agona and *S*. Dublin) were sensitive/intermediate to all tested antibiotics. In total, 25.49% (13/51) and 11.76% (6/51) of isolates resulted in a resistance to 1 and 2 antibiotics, respectively. A considerable number of strains showed multiple antimicrobial resistance, as follows: in total, 5.88% (3/51) were resistant to 3 antimicrobials, an additional 5.88% (3/51) were resistant to 4 antimicrobials, and 47.06% (24/51) were resistant to 5 or more antimicrobials.

**Figure 3:**
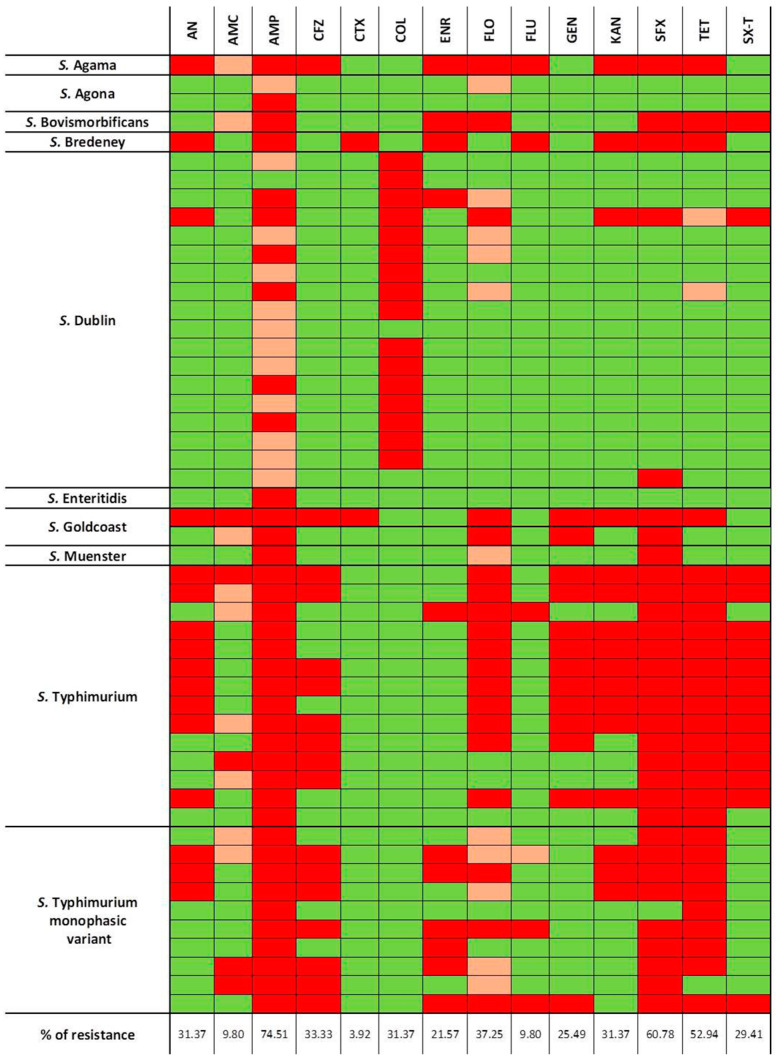
The phenotypic antibiotic resistance profiles of *Salmonella* spp. isolates analyzed using the MIC assay, divided by serotypes. The color codes indicate resistant (red), intermediate (orange), and susceptible (green) phenotypes to 14 antibiotics. Antibiotics abbreviations: SX-T: Trimethoprim + Sulphonamides; TET: Tetracycline; SFX: Sulfisoxazole; KAN: Kanamycin; GEN: Gentamicin; FLU: Flumequine; FLO: Florfenicol; ENR: Enrofloxacin; COL: Colistin; CTX: Cefotaxime; CFZ: Cefazolin; AMP: Ampicillin; AMC: Amoxicillin + Clavulanic Acid, AN: Aminosidine.

The most common antimicrobial for which *Salmonella* isolates were resistant was ampicillin (74.51%, 38/51), followed by sulfisoxazole (60.78%, 31/51), tetracycline (52.94%, 27/51), and florfenicol (37.25%, 19/51). Of note, colistin showed a resistance rate of 31.37% (16/51). The antibiotic with the lowest rate of resistance was cefotaxime (3.92%, 2/51) (Figure 3).

**Figure 4:**
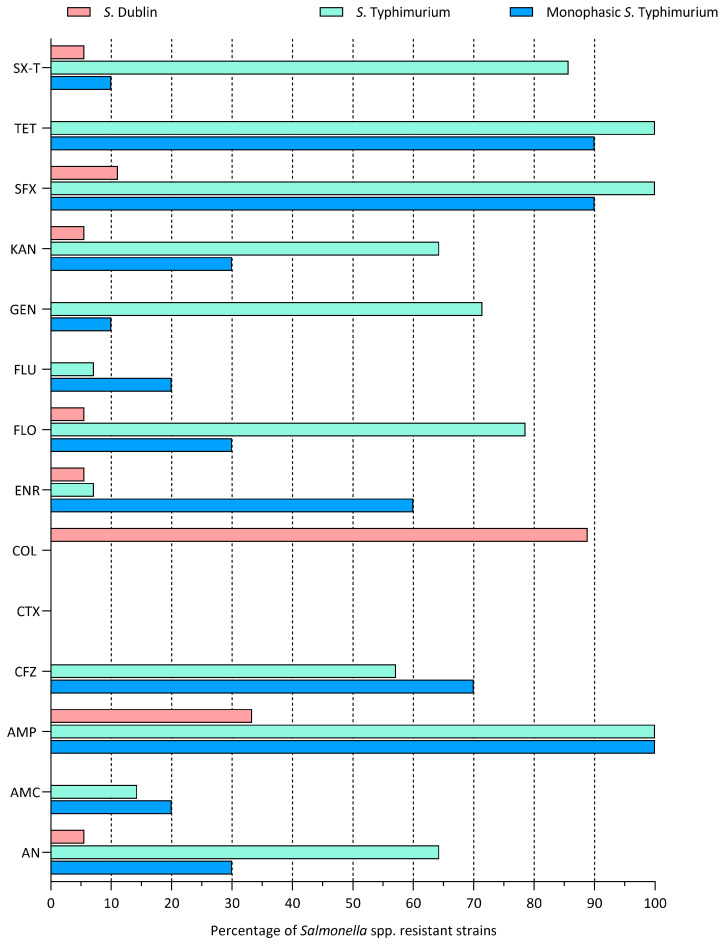
Occurrence of resistance to 14 selected antibiotics in *Salmonella* Dublin (pink), Typhimurium (light blue), and its monophasic variant (blue) isolates. Antibiotics abbreviations: SX-T: Trimethoprim + Sulphonamides; TET: Tetracycline; SFX: Sulfisoxazole; KAN: Kanamycin; GEN: Gentamicin; FLU: Flumequine; FLO: Florfenicol; ENR: Enrofloxacin; COL: Colistin; CTX: Cefotaxime; CFZ: Cefazolin; AMP: Ampicillin; AMC: Amoxicillin + Clavulanic Acid, AN: Aminosidine.

Out of the 51 isolates tested for susceptibility to antibiotics, 18, 14, and 10 were characterized as *S*. Dublin, *S*. Typhimurium, and its monophasic variant, respectively. *S*. Typhimurium and its monophasic variant reported a high percentage of resistance to ampicillin (100%) and tetracycline (100% and 90%, respectively). On the other hand, 0% of *S*. Typhimurium and its monophasic variant were resistant to colistin. Regarding *S*. Dublin, a higher resistance rate was observed for colistin (88.89%). Of interest, this serotype reported resistance rates of 33.33% and 0% for ampicillin and tetracycline, respectively (Figure 4).

### 3.7. Autogenous Vaccine Administration

For 15 farms with *Salmonella* clinical disease, field veterinary practitioners prescribed the administration of an autologous vaccine. The number of farms vaccinated with autogenous vaccines and the different *Salmonella* serotypes present in the vaccine are summarized in Table 5.

## 4. Discussion

Bovine salmonellosis in dairy cattle poses important risks both because it represents a food safety problem and has important animal health implications [7,26,27]. In addition, *Salmonella* outbreaks in dairy cattle herds result in significant productive and economic losses for farmers due to missed benefits (e.g., discarded milk or reduced milk yield due to disease and neonatal mortality), costs to treat/control the infection [28,29], and possible restrictions imposed by authorities. Our study deals with the detection of *Salmonella* spp. in dairy cattle farms, focusing on its isolation from different sources and the antimicrobial resistance profile of the isolates. For this purpose, we retrospectively analyzed 120 cases of *Salmonella* spp. infections that occurred in the Cremona and Mantua provinces in the period from 2021 to 2022. 

In our study, the *S*. *enterica* serovars most frequently isolated in dairy cattle farms were *S*. Dublin, *S*. Typhimurium, and the *S*. Typhimurium monophasic variant. This result is consistent with the prevalence data of *Salmonella* serotypes in cattle reported in Italy [30] and in Europe [7]. The *S*. Typhimurium serotype affects a broad spectrum of animal species and presents a cosmopolitan distribution [31], whereas *S.* Dublin is the host-adapted serotype in cattle, occurring worldwide, with estimates of the proportion of infected farms varying greatly among countries [4]. The finding that the majority (61.67%) of the first detection of *Salmonella* spp. was in calf carcasses/organs conferred to the IIZZSS Laboratories for diagnostic purposes is justified by the fact that *Salmonella* is a frequent cause of mortality among calves, associated with clinical signs such as diarrhea, fever, and, to a lesser extent, respiratory and nervous signs. In a lower number of cases (13.33%), the first *Salmonella* isolation in the herd was from feces and rectal swabs, taken because of enteric clinical signs in calves. This type of sample is chosen both in the early stages of *Salmonella* infection, when mortality is not yet present, and because it is more convenient than sampling organs/carcasses. In 21.67% of the cases included in this study, *Salmonella* was isolated from the livers of adult cattle that were collected during the emergency slaughters conducted outside the slaughterhouses. Post-mortem inspections and sample collections were carried out by the official veterinarians. 

The macroscopic lesions observed in calves during autopsy were suggestive of salmonellosis. In accordance with other studies, enteritis, observed in 68 out of 91 calves, was the most common pathological finding [32,33], followed by hepatosplenomegaly (29/93) and pneumonia (23/91). Considering the different *Salmonella* serotypes isolated in the present study from calves, *S*. Dublin, *S*. Typhimurium, and its monophasic variant were the most represented with, respectively, 29, 27, and 21 isolates.

It is well known that *S*. Dublin is a concern for the dairy industry, because it is highly adapted to cattle and it is frequently associated with enteritis and septicemia. In addition, this serotype can be responsible for respiratory diseases in calves with inflammatory lesions in the lungs [6,34]. Consistently, in our study, in *S*. Dublin-infected animals, hepatosplenomegaly, suggestive of a septicemic form, was the most frequently observed lesion (15/29), followed by enteritis (14/29) and pneumonia (9/29).

In the *S*. Typhimurium and its monophasic variant-infected animals, the majority of cases (42/48) reported enteritis, while hepatosplenomegaly and pneumonia were reported in 13/48 and 12/48 cases, respectively. These lesions are frequently reported in *S*. Typhimurium and its monophasic variant infection in calves [33,34]. 

Once *Salmonella* has been identified on a farm, control measures have been communicated to the farmer and field and official veterinarians to reduce the spread of the infection. Some of these measures included the separation of affected animals and the destruction of their milk; therapeutic treatments; extraordinary cleaning and disinfection sessions of the individual/collective pens, maternity areas, and all the equipment; improvement of management measures such as cleaning and disinfection between the individual occupied pens, leaving the pens empty for several days; cleaning and disinfection procedures for validation with surface samplings; maintenance of clean and dry litter by the restoring, leveling, and rearranging of the material; management of the milking operations and udder hygiene; intensification of the pest control plan; and restrictions of animal movements to/from the herd. The restrictions were lifted when cleaning and disinfection were completed and there were no clinical signs of infection in the herd. 

Among the farms where further *Salmonella* analyses were carried out, the results of *Salmonella* fecal shedding in calves were helpful in improving calving area management with the identification and separation of *Salmonella* shedders, which is an important control measure to reduce the possibility of dispensing *Salmonella* within the calving area environment [3]. In addition, the movement of male calves to fattening herds was allowed only after negative results for *Salmonella* was obtained. Indeed, movement restrictions are a key measure to control the transmission of the pathogen between herds.

With respect to the environmental sampling, since the contaminated area should be the main focus for concentrating cleaning and disinfection efforts, environmental samples were taken from high-risk housing areas, such as the group maternity areas and collective pens for calves. In a study by Fossler et al. (2005), maternity and sick pens were recognized as more likely to be *Salmonella* positive than other environmental locations [35]. In our study, 100 absorbent overshoes from 16 farms were analyzed for *Salmonella* spp., revealing a proportion of positivity of 34/100. As expected, *Salmonella*-positive cultures were observed both in collective pens for calves (22/61) and in maternity housing (12/33). The maternity areas were shared with multiple periparturient cows at the same time, increasing the possibility of *Salmonella* contamination of the environment. *Salmonella* can be spread in the environment by the feces of *Salmonella*-shedding animals, which may be asymptomatic carriers. Therefore, the good management and hygiene of these farm areas is crucial to limit cross-infection among calves housed in collective pens and to reduce exposure to *Salmonella* in newborn calves in the maternity area, where maternity beds are their first point of contact with the environment. Maintaining a clean and dry substrate is known to be a preventive measure that potentially reduces the risk of disease for calves and cows [36]. 

Of note, the effectiveness of cleaning and disinfection procedures in place on the farm was assessed via the post-disinfection sampling of individual calf pens, using surface swabs or sponges. *Salmonella* spp. were recovered in 9 out of 27 environmental samples (4/7 farms). In these farms with positive results, the cleaning and disinfection protocol and its application were verified and optimized. The use of high-power washing was discouraged, because although efficient in removing organic debris, it has the disadvantage of creating an aerosol that can spread *Salmonella* in the environment. Implementing proper cleaning and disinfection procedures for calf pens and all equipment is important to reduce the chances of *Salmonella* infection for both animals and humans. 

Of note, 93 bulk tank milk samples were collected from 32 farms, of which three samples (from three farms) yielded a *Salmonella* spp. positive result. *Salmonella*-positive bulk milk is most likely due to the fecal contamination of milk rather than true lactational shedding, because *Salmonella* mastitis is an extremely rare event [3]. The presence of *Salmonella* in bulk milk poses a potential risk for consumers of raw and unpasteurized milk. Thus, the improvement of the optimal management of milking operations, udder hygiene, and accurate cleaning and disinfection procedures for milk tanks are critically important in order to prevent the *Salmonella* contamination of bulk milk and to mitigate the risk for consumers. The eventual distribution of raw milk for human consumption, which is currently an uncommon practice in the studied area and still involves boiling raw milk before consumption, is promptly suspended in the case of *Salmonella* outbreaks in dairy farms. 

The administration of an autogenous vaccine is a possible strategy to assist the control of *Salmonella* in dairy farms and is a promising alternative to the use of antimicrobials. Few scientific studies on the efficacy of autogenous vaccines for *Salmonella* are available; in a recent study, unvaccinated feedlot cattle housed adjacent to or with vaccinated animals seem to benefit from the protection of vaccinated animals in mitigating *Salmonella* in lymph nodes [37].

In our study area, there has been an increase over time in the demands for the production of autogenous vaccines for *Salmonella*. Interestingly, in one farm, the vaccine developed contained the three different strains of *Salmonella* isolated in the herd—*S*. Typhimurium, *S*. Typhimurium monophasic variant, and *S*. Agama. In another case, the vaccine produced contained two isolated strains—*S*. Dublin and *S*. Typhimurium. An advantage of autogenous vaccines is that they can contain more than one isolated strain circulating in the herd, resulting in a high specificity for the farm. Since most of the clinical cases involved calves, cows at dry-off were vaccinated in order to stimulate the production of *Salmonella* antibodies that are passed on to the calf through colostrum intake. Passive immunity stimulated by the *Salmonella* vaccination of dry cows was previously reported [38]. When clinical signs were observed in the postweaning period, the vaccination was extended to other categories, such as adult cattle, heifers, and post-weaned calves. Adverse reactions to the vaccine, such as anaphylactic reactions, did not occur in any of the farms. 

Since this is an autogenous vaccine, its use is not allowed to continue indefinitely, as required by current regulations. During the period 2021–2023, after vaccination was discontinued, *Salmonella* infection recurred in a single herd, with an infection of the same serotype (*S*. Typhimurium monophasic variant) one year after vaccination ended. Our study does not provide data about the efficacy of autogenous vaccines in conferring protection against *Salmonella* infection, and further studies on this aspect are needed, as this approach holds promise for *Salmonella* control in dairy herds. In any case, the use of vaccination is an additional component of *Salmonella* control, although it does not replace good hygiene practices and management, as well as all instituted control measures. 

Regarding antibiotic resistance, 51 non-repetitive *Salmonella* isolates were characterized using the MIC assay to determine antibiotic susceptibility. Our results highlighted the circulation in dairies of multi-drug resistant *Salmonella* strains with nearly 50% of isolates resistant to 5 or more antimicrobial classes. The overall resistance rate observed to ampicillin, sulfisoxazole, and tetracycline was high with 74.51%, 60.78%, and 52.94% of the isolates. The tetracycline and sulfisoxazole resistance rates are higher than that reported in 2021 in Europe (respectively, 36.7% and 39.2%), but are consistent with the Italian data (60%). The resistance to ampicillin observed in the present study (74.51%) is higher than that reported by EFSA in Europe (22.8%) and Italy (35%) in 2021 [39]. The difference in resistance pattern might be attributed to different farm management methods, such as different antibiotic treatment choices, which can select for AMR. Moreover, *S*. Typhimurium and its monophasic variant, which have been reported to show high resistance rates of ampicillin and tetracycline [39,40,41], are highly represented in this analysis (24/51). 

In the present study, in accordance with what was reported in Europe in 2021, a low rate of resistance to cefotaxime (recognized as one of the highest priority critically important antibiotics—HPCIAs) was observed (3.92%) [39]. Of interest, the two cefotaxime resistant strains reported belonged to serotypes Goldcoast and Bredeney. Another antibiotic categorized as an HPCIA is colistin, which is a last-resort treatment in humans for healthcare-associated infections sustained by carbapenemase-producing Gram-negative bacteria. The overall colistin resistance observed in the present study is 31.37% (16/51). This value is much higher compared to that reported in Europe in 2021 (11.4%), but this discrepancy could be explained, given that all the studied colistin-resistant isolates were *S*. Dublin (16/16). It is known that this serotype, belonging to group D *Salmonella* (serogroup O9), shows a decreased susceptibility to colistin, although no acquired or mutational colistin resistance mechanisms are present [42,43]. 

Although the antimicrobial treatment of salmonellosis is controversial, it is sometimes justified in severe clinical cases. The prudent use of antimicrobials is recommended by the scientific and medical communities with an emphasis on the need for susceptibility testing of *Salmonella* strains, avoiding the metaphylaxis approach, and using the narrowest spectrum of antibiotic indicated by susceptibility data, to preserve the efficacy of the available antimicrobials.

Further studies should be carried out for relating *Salmonella* strains isolated in the dairies and assessing genotypic profiles of antimicrobial susceptibility with the aid of WGS technologies.

## 5. Conclusions

Our findings indicate that in dairy farms of the Cremona and Mantua provinces (North Italy), which play a significant role in milk production in Italy, *Salmonella* was most commonly identified first from calf carcasses and organs. The serotypes most frequently detected were *S*. Dublin, *S*. Typhimurium, and the *S*. Typhimurium monophasic variant. The isolation of *Salmonella* from various sources and environmental samples is evidence of the wide distribution of the bacterium on the farm. Salmonellosis in dairy cattle farms can have an impact on animal health, welfare, and production costs, and poses a zoonotic risk. Thus, during a *Salmonella* outbreak, control strategies should be rapidly implemented, including the treatment, isolation of infected animals, and application of proper hygiene measures such as the cleaning and disinfection of housing areas and equipment to decrease the risk of disease transmission to both humans and cattle. Finally, the antibiotic resistance profiles of *Salmonella* spp. reported in the present study can be useful to improve the knowledge of multi-drug resistant strains circulating in North Italian dairy farms and to develop effective on-farm antimicrobial stewardship practices.

## Figures and Tables

**Table 1:** Number and proportion (%) of *Salmonella* serovars identified in dairy cattle farms in the period 2021–2022.

*Salmonella enterica* susp. *enterica* Serovars	*N*° of Cases	Prevalence (%)
*S.* Dublin	46	38.33
*S*. Typhimurium	28	23.33
*S*. Typhimurium monophasic variant	17	14.17
*Salmonella* spp.	2	1.67
*S.* London	2	1.67
*S*. Agona	2	1.67
*S*. group O:9 (D1)	2	1.67
*S*. Muenster	2	1.67
*S*. Bredeney	1	0.83
*S*. Chester	1	0.83
*S.* Infantis	1	0.83
*S.* Goldcoast	1	0.83
*S*. Bovismorbificans	1	0.83
*S.* Enteritidis	1	0.83
*S*. Dublin and *S*. Typhimurium	3	2.50
*S*. Dublin and *S*. Goldcoast	2	1.67
*S*. Typhimurium and *S*. Bredeney	1	0.83
*S*. Newport and *S*. Kentucky	1	0.83
*S*. Bovismorbificans and *S*. Infantis	1	0.83
*S*. Dublin and *S*. Bredeney	1	0.83
*S*. Typhimurium and *S*. Senftenberg	1	0.83
*S*. Typhimurium and *S*. Typhimurium monophasic variant	1	0.83
*S*. Agama, Typhimurium, and *S*. Typhimurium monophasic variant	1	0.83
*S*. Dublin, *S*. Typhimurium, and *S.* Typhimurium monophasic variant	1	0.83
Total	120	100
*Salmonella* co-presence	13	10.83

**Table 2:** Macroscopic lesions detected in 91 calves subjected to autopsy and positive for *Salmonella* spp.

*Salmonella* Serovars	*N*° Herds	*N*° Positive Calves	Enteritis	Pneumonia	Hepatosplenomegaly
*S*. Agama	1	1	1	1	0
*S*. Agona	2	2	2	1	0
*S*. Bovismorbificans	1	2	2	0	0
*S*. Bredeney	2	3	3	0	0
*S*. Dublin	24	29	14	9	15
*S*. group O:9 (D1)	2	2	2	0	1
*S*. Goldcoast	1	1	1	0	0
*S*. Muenster	2	3	1	0	0
*S*. Typhimurium	18	27	22	7	12
*S*. Typhimurium monophasic variant	11	21	20	5	1

**Table 3:** The table shows the analyses performed in the dairy cattle herds after first *Salmonella* detection.

Samples Analyzed	*N*° Farms with Positive Samples/*N°* Total Farms Tested	*N*° Positive Samples/*N°* Total Samples
Feces/Rectal swabs	26/50	206/2145
Bulk milk	3/32	3/93
Calf carcasses/organs	15/21	21/36
Environmental overshoes	7/16	34/100
Environmental swabs/Sponges	4/7	9/27
Fetuses	2/3	3/4

**Table 4:** Distribution of *Salmonella enterica* isolates tested for antibiotic resistance according to their serovar.

*Salmonella enterica* subsp. *enterica* Serovars	*N*° of Tested Isolates
*Salmonella* Dublin	18
*Salmonella* Typhimurium	14
*Salmonella* Typhimurium monophasic variant	10
*Salmonella* Agona	2
*Salmonella* Goldcoast	2
*Salmonella* Agama	1
*Salmonella* Bovismorbificans	1
*Salmonella* Bredeney	1
*Salmonella* Muenster	1
*Salmonella* Enteritidis	1
Total	51

**Table 5:** Number of farms vaccinated with an autogenous vaccine and *Salmonella* serovar(s) contained in the vaccine.

*N°* of Farms	*Salmonella* Serovars Present in the Vaccine
6	*S*. Dublin
3	*S*. Typhimurium
3	*S*. Typhimurium monophasic variant
1	*S*. Muenster
1	*S*. Typhimurium monophasic variant, *S*. Agama, and S. Typhimurium
1	*S*. Dublin and *S*. Typhimurium

## Data Availability

Data are contained within the article.
